# The emerging role of the MiR-1272-ADAM9-CDCP1 signaling pathway in the progression of glioma

**DOI:** 10.18632/aging.202196

**Published:** 2020-11-26

**Authors:** Fei Geng, Gui-Feng Lu, Yu-Jun Luo, Sky Dominguez, De-Ying Kong, Lian-Hua Shen, Xiao-Mei Luo, Xin Yang, Min Hu, Wen-Shan Lai, Zhi-Shui Jiang, Yuan-Shou Chen

**Affiliations:** 1 Department of Physiology, Zunyi Medical University, Zunyi, China; 2 Department of Pathophysiology, Zunyi Medical University, Zunyi, China; 3 Rehabilitation Department, Hubei Provincial Hospital of Traditional Chinese Medicine, Hubei Province Academy of Traditional Chinese Medicine, Wuhan, China; 4 Department of Psychiatry and Behavioral Sciences, Northwestern University Feinberg School of Medicine, Chicago, IL 606011, USA

**Keywords:** miR-1272, ADAM9, CDCP1, glioma

## Abstract

Glioma is a primary, malignant, and aggressive brain tumor in adults. To develop new therapeutic strategies for glioma, we must determine its underlying mechanisms. In the present study, we aimed to investigate the potential role of miR-1272-ADAM9-CDCP1 signaling in the progression of glioma. We found that ectopic expression of miR-1272 produced significant inhibitory effects on cell proliferation and migration and was associated with cell cycle G0/G1 arrest in A172 and SHG44 glioma cells. Using the luciferase reporter assay, we identified ADAM9 as a target of miR-1272. The expression of ADAM9 was markedly decreased or increased after overexpression or inhibition, respectively, of miR-1272 in glioma cells. Moreover, overexpression of ADAM9 reversed the inhibitory effects of miR-1272 on glioma cell progression. Furthermore, CDCP1 served as a potential downstream molecule of miR-1272/ADAM9 signaling in glioma and promoted the proliferation and migration of glioma. Results derived from clinical samples and online databases confirmed correlations between the expression of ADAM9 and CDCP1 and both the severity and prognosis of glioma. In conclusion, these results suggest that miR-1272 and CDCP1 may act as novel regulators in glioma. The miR-1272/ADAM9/CDCP1 pathway may serve as a potential candidate pathway for the prevention of glioma.

## INTRODUCTION

Glioma is an aggressive brain cancer that accounts for the majority of malignant tumors found in the human brain [1]. Glioma is categorized as a grade I-IV tumor [2], according to the World Health Organization (WHO) guidelines, depending on the cells involved and the expression of key mutations within tumor cells. Despite the current treatment options, including chemotherapy, radiation therapy, and surgical resection, the prognosis of most glioma patients remains poor [3, 4]. To understand glioma tumorigenesis and to develop novel treatment therapies, we must first investigate the underlying molecular mechanisms of glioma. This paper will determine what roles microRNAs (miRNAs) may play in glioma formation and progression, as miRNAs are already used to diagnose tumors in clinical practice [5].

MiRNAs are a set of non-coding and single RNA molecules. They play an important role in the modulation of downstream gene expression via binding to the 3’-UTR of target genes, giving rise to translation suppression or mRNA degradation [6]. Many scientists believe that miRNAs also have important roles in human tumors due to the oncogenic and suppressive roles of their target molecules [5]. In recent years, overwhelming evidence has suggested that the dysregulation of miRNAs is indeed linked with cell proliferation, cell migration, cell apoptosis, and cell cycle progression, metabolism, and differentiation [7, 8]. Accumulating studies have identified a number of miRNAs that are involved in brain tumors and other neural diseases; these miRNAs are potential targets for future studies [9–11].

Previous studies have found that miRNAs function as tumor suppressors [12, 13] and tumor oncogenes [14] in glioma. However, more individual miRNAs require further study to determine their specific effects. One previous study reported that the expression level of the miRNA miR-1272 was higher in patients with narcolepsy [15]. In our preliminary study, we found that the expression of miR-1272 was decreased in glioma. Based on this finding, we decided to further study the role and mechanisms of miR-1272 in glioma.

In the current study, we investigated the role of a novel microRNA, miR-1272, in the progression of glioma. We sought to address the following questions: (I) What roles do miR-1272 play in tumor progression? (II) What is the potential direct target of miR-1272? (III) Does miR-1272 overexpression cause inhibition of cell proliferation, inhibition of migration, promotion of apoptosis, or cell cycle arrest via its downstream pathway? (IV) What are the clinical implications of miR-1272 and its target molecules in clinical specimens? In general, the answers to these questions may provide new insights into glioma and will help establish a unique miRNA-1272-based therapy or diagnosis for glioma.

## RESULTS

### MiR-1272 prohibits tumor proliferation and migration in glioma cells

**Figure 1:**
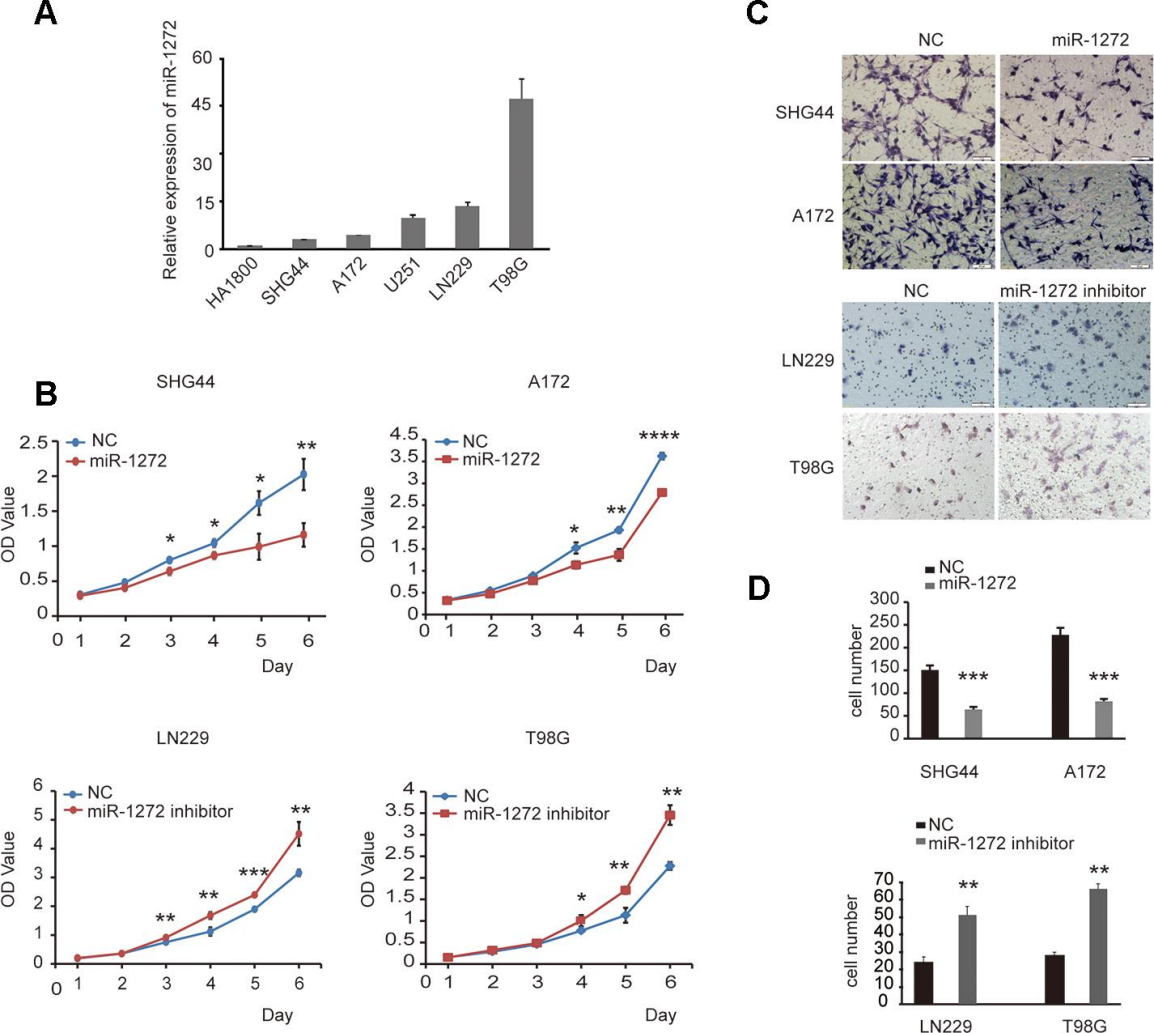
**MiR-1272 prohibits tumor proliferation and migration in glioma cells.** (**A**) The expression of miR-1272 in HA1800, SHG44, A172, U251, LN229, and T98G cell lines was detected by qPT-PCR. (**B**) CCK-8 assay was applied to explore the effects of miR-1272 on the proliferation of glioma cells transfected with miR-1272 mimic or inhibitor. (**C** and **D**) Ectopic expression of miR-1272 inhibited the migration of glioma cells. Error bars represent mean ± SEM. NC, negative control. * p < 0.05, ** p < 0.01, *** p < 0.001.

To confirm the role of miR-1272 in glioma progression, endogenous expression of miR-1272 was assessed in a number of glioma cell lines and normal human astrocyte cells. The results showed that the expression of miR-1272 in the SHG44 and A172 lines was low, while LN229 and T98G cells exhibited strong expression of miR-1272 (Figure 1A). Furthermore, cell proliferation, migration, and the cell cycle stages were analyzed. Cells transfected with miR-1272 mimics and NC were collected and significant overexpression of miR-1272 in SHG44 and A172 cells was detected (Supplementary Figure 1A). Cell viability was significantly decreased in cells transfected with miR-1272 mimics compared with miR-NC transfected cells (Figure 1B). A Transwell migration assay was used to assess the roles of miR-1272 in migration of SHG44 and A172 cells. The results indicated that ectopic expression of miR-1272 decreased the migrating ability of SHG44 and A172 cells (Figure 1C, 1D).

The above results demonstrate that ectopic expression of miR-1272 inhibits the progression of glioma. To confirm if inhibition of miR-1272 has the opposite effect, the miR-1272 inhibitor and its negative control were prepared and used to transfect T98G and LN229 cells (Supplementary Figure 1B). Cell viability was significantly increased in cells transfected with miR-1272 inhibitors (Figure 1B). Knockdown by the miR-1272 inhibitor exhibited a weaker ability to migrate from the upper chamber to the lower chamber in LN229 and T98G cells (Figure 1C, 1D).

Collectively, the in vitro results demonstrate that miR-1272 prohibits tumor growth and migration, thus playing a tumor suppressive role in the progression of glioma.

### Cell cycle arrest is induced by miR-1272 in glioma cells

**Figure 2:**
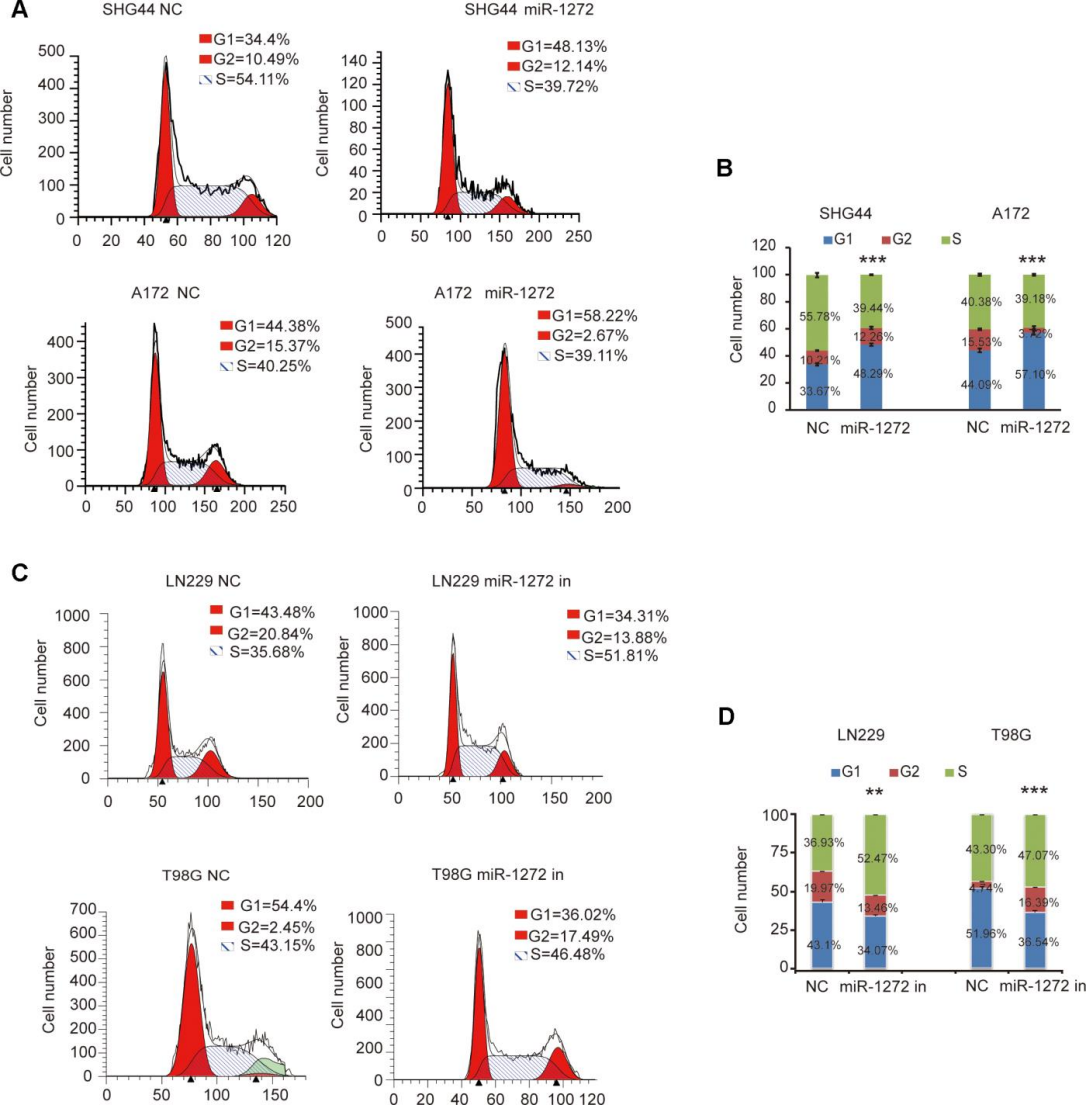
**Cell cycle arrest is induced by miR-1272 in glioma cells.** (**A** and **B**) MiR-1272 induced glioma cell cycle arrest, as assessed by flow cytometry analysis. (**C** and **D**) Flow cytometry was used to detect the cell cycle distribution of LN229 and T98G cells with or without miR-1272 knockdown. Error bars represent mean ± SEM. Statistical results of G1 phase were marked with asterisks. ** p < 0.01, *** p < 0.001.

To further confirm the influence of miR-1272 on glioma cell cycle stages, cell cycle analyses were performed after ectopic expression or knockdown of miR-1272 in glioma cells. The results indicated that miR-1272 increased the number of cells accumulating in the G1 phase (Figure 2A, 2B). Moreover, the percentages of glioma cells in the S and G2 phases were decreased (Figure 2A, 2B). In contrast, cell cycle arrest was abolished in cells treated with miR-1272 inhibitor, with less cells in the G1 peak and more cells in the S and G2 phases (Figure 2C, 2D). These results clearly demonstrate that miR-1272 may function as a tumor suppressor by inducing cell cycle arrest.

### MiR-1272 directly targets ADAM9 and regulates its expression in vitro

**Figure 3:**
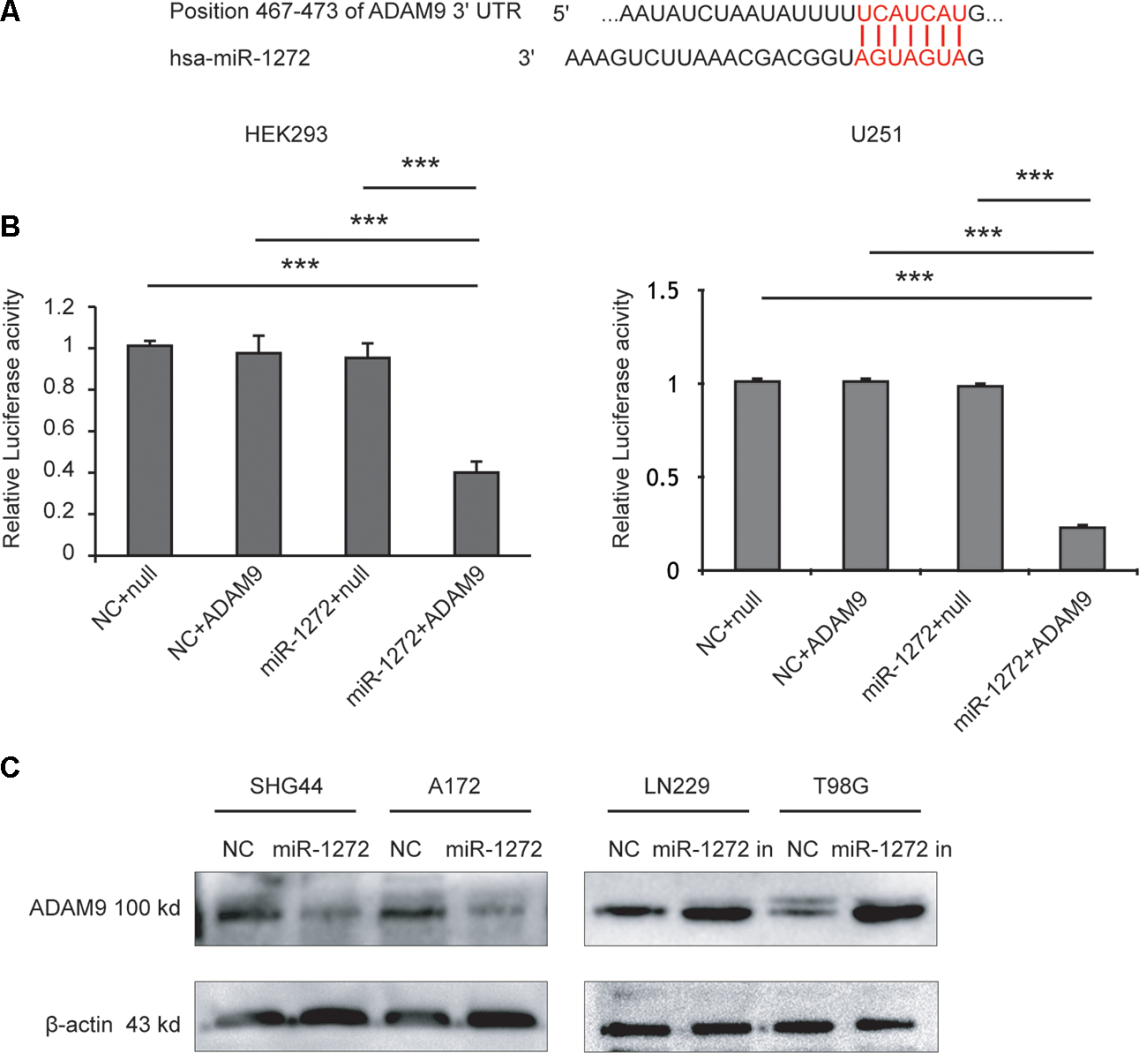
**MiR-1272 directly targets ADAM9 and regulates its expression in vitro.** (**A**) The binding sites of miR-1272 and ADAM9. (**B**) Luciferase activities decreased upon transfection with miR-1272 and ADAM9 3'-UTR luciferase reporter vector in HEK-293 and U251 cells. (**C**) MiR-1272 regulated the protein expression of ADAM9 detected by western blotting. Null, psiCHECKTM-2 report plasmid without ADAM9 3'-UTR. Error bars represent mean ± SEM. *** p < 0.001.

To explore the tumor suppressive molecular mechanisms of miR-1272, candidate miR-1272 target genes were analyzed using multiple prediction algorithms (miRBase, PicTar, and TargetScan). These computations predicted a series of potential target genes, identifying one potential miR-1272 binding site in the 3’-UTR of ADAM9 mRNA (Figure 3A). To investigate whether miR-1272 directly targets ADAM9, we performed a luciferase reporter assay. ADAM9 3’-UTR fragments were cloned downstream of the firefly luciferase reporter gene. The luciferase activity was decreased when cells were co-transfected with miR-1272 mimics and luciferase reporter vectors; however, co-transfection of miR-1272 mimics and control reporter plasmids did not affect the luciferase activity in HEK293 and U251 cells (Figure 3B). These results indicate that miR-1272 can bind to the ADAM9 3’-UTR.

Then, to determine whether miR-1272 could repress this putative target, glioma cells were transfected with miR-1272 mimic or inhibitor. Following transfection, protein levels of the target gene were assessed using western blotting analysis. Increased miR-1272 expression remarkably reduced ADAM9 expression in SHG44 and A172 cells, while miR-1272-inhibitor-transfected cells had higher protein levels of ADAM9 (Figure 3C). These findings indicate that miR-1272 negatively regulates ADAM9 expression in glioma cells.

### Ectopic expression of ADAM9 abolishes miR-1272-induced anti-tumor effects on glioma cell behaviors

**Figure 4:**
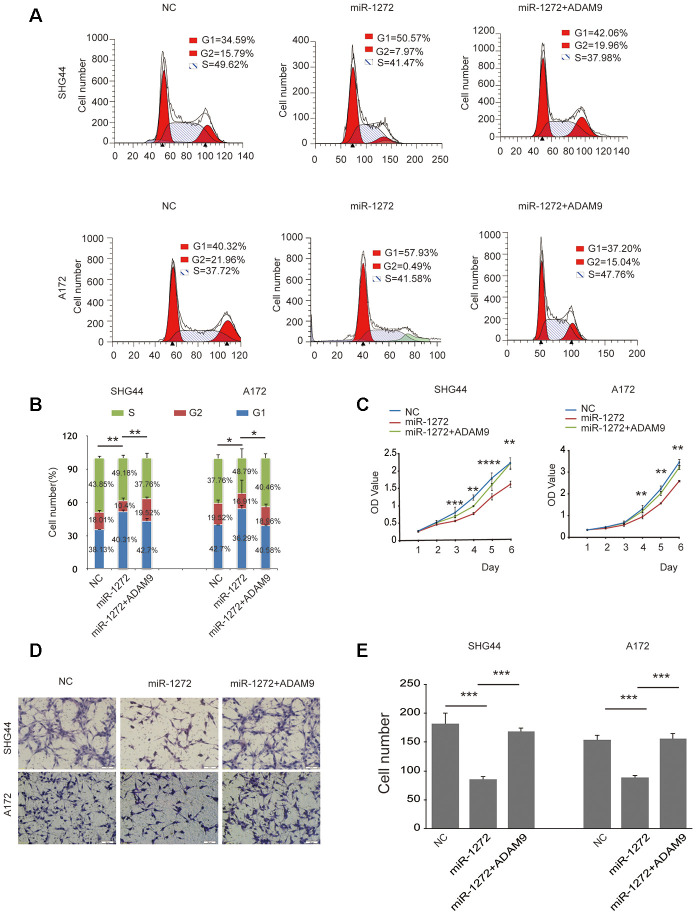
**Ectopic expression of ADAM9 abolishes miR-1272-induced anti-tumor effects on glioma cell behaviors.** (**A**) ADAM9 re-shaped the glioma cell cycle distribution induced by miR-1272, as assessed with flow cytometry. (**B**) Statistical diagram of the cell cycle results. Statistical results of G1 phase were marked with asterisks. (**C**) Restoration of ADAM9 reversed miR-1272-induced effects on cell proliferation in glioma cells, as assessed by proliferation assay. (**D**) ADAM9 abolished miR-1272-induced inhibition of cell migration, as assessed by Transwell assay. (**E**) Statistical diagram of the migration results. Error bars represent mean ± SEM. * p < 0.05, ** p < 0.01, *** p < 0.001.

To determine whether miR-1272 inhibits cell growth and migration by targeting the ADAM9 encoding gene, we performed rescue experiments in which we restored ADAM9 expression (Supplementary Figure 1C) in cells transfected with miR-1272 mimic. Ectopic expression of ADAM9 decreased the number of cells in the G1 phase induced by miR-1272 (Figure 4A, 4B). Restoring the expression of ADAM9 overwhelmingly reversed the suppression of cell proliferation by miR-1272 (Figure 4C). Furthermore, ADAM9 remarkably prohibited the suppression of cell migration induced by miR-1272 (Figure 4D, 4E) in SHG44 and A172 cells. These results further demonstrate that miR-1272 suppresses proliferation and migration while promoting apoptosis by targeting ADAM9.

### MiR-1272 is downregulated in glioma tissues and is negatively correlated with ADAM9

**Figure 5:**
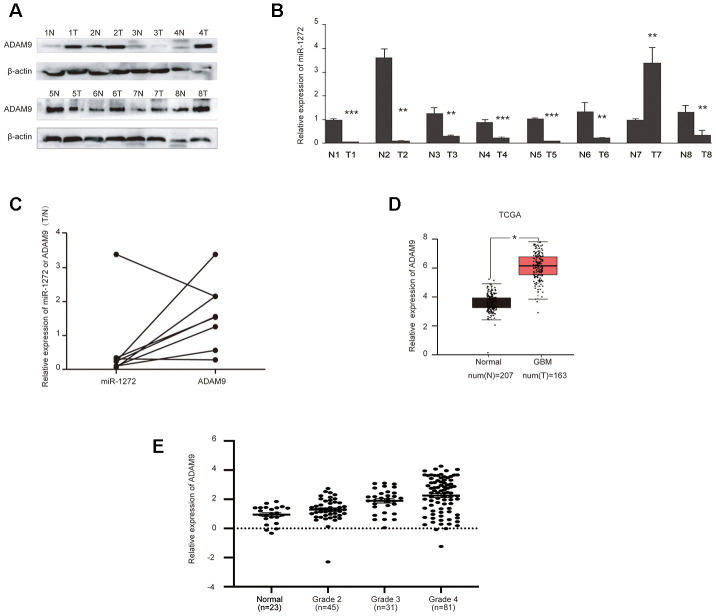
**MiR-1272 is downregulated in glioma tissues and is negatively correlated with ADAM9.** (**A**) ADAM9 showed increased expression in glioma tissues compared with their adjacent tissues, as assessed by western blotting. (**B**) Relative expression of miR-1272 in glioma tissues, as assessed by qRT-PCR. (**C**) Opposite expression patterns of miR-1272 and ADAM9 in clinical glioma tissues. (**D**) Upregulation of ADAM9 in clinical glioma tissues derived from the TCGA database. (**E**) ADAM9 expression in clinical glioma tissues was associated with malignant grade. Error bars represent mean ± SEM. * p < 0.05, ** p < 0.01, *** p < 0.001.

To ascertain the clinical relevance of miR-1272 as a tumor suppressor, the expression levels of miR-1272 and its target gene in glioma tissues were assessed by qRT-PCR and western blotting analyses. RNA samples and tissue extracts were collected from eight glioma tissues and eight matched tissues. Western blot analyses showed higher ADAM9 expression in glioma tissues compared to normal tissues (Figure 5A). Accordingly, miR-1272 expression was remarkably lower in glioma tissues (Figure 5B). Thus, inverse expression patterns of miR-1272 and ADAM9 in glioma tissues were observed (Figure 5C), supporting our previous findings.

Due to our limited number of samples, ADAM9 expression data in glioma were obtained from the TCGA database. In these samples, we found that ADAM9 was upregulated in glioblastoma (Figure 5D). Further analysis of the association between ADAM9 expression and tumor grade of glioma patients revealed a significant association between ADAM9 expression and glioma malignant grade (p = 0.001, Figure 5E), which indicates that ADAM9 is correlated with the severity of glioma. More importantly, despite the significant association between ADAM9 expression and disease-free survival (Supplementary Figure 2B), the high expression of ADAM9 was not significantly associated with overall poorer prognosis in patients diagnosed with glioma (Supplementary Figure 2A). Taken together, these results reveal reversed expression patterns between miR-1272 and ADAM9 and indicate that the expression of ADAM9 is associated with the severity of glioma.

### MiR-1272 functions as a glioma repressor through the ADAM9-CDCP1 pathway

**Figure 6:**
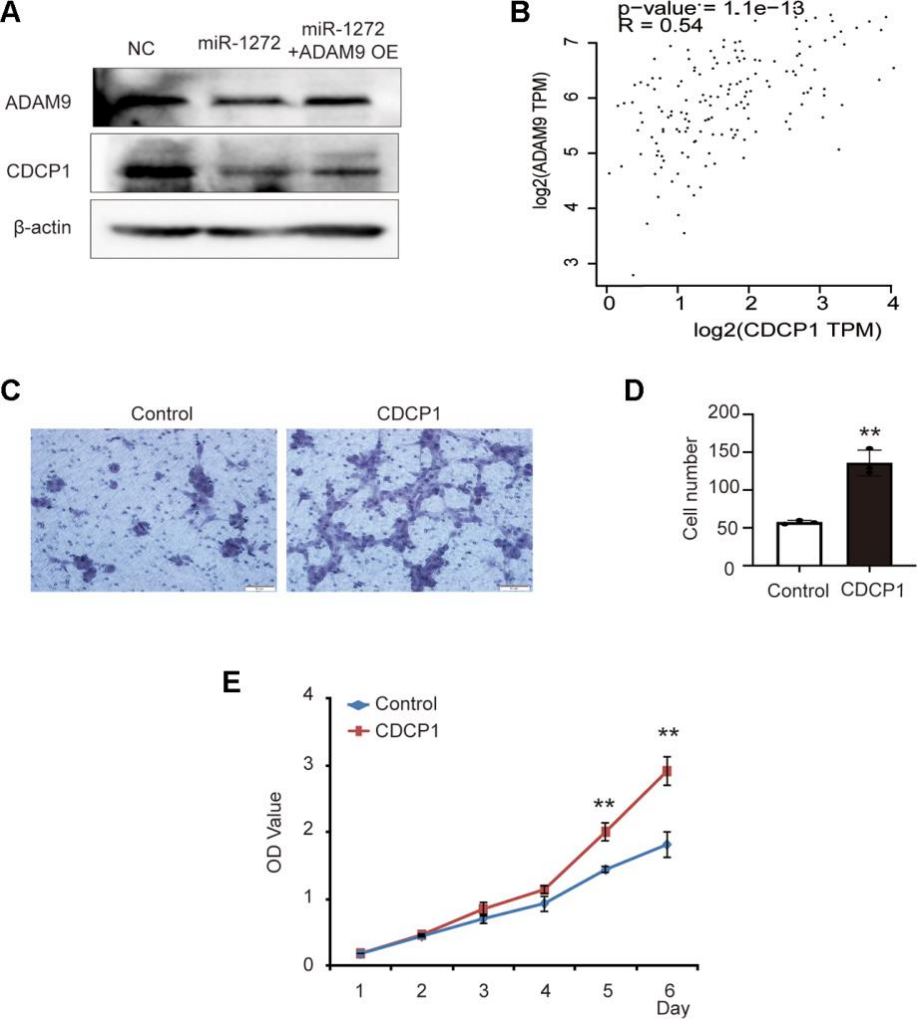
**MiR-1272 functions as a glioma repressor through the ADAM9-CDCP1 pathway.** (**A**) Relative expression of ADAM9 and CDCP1 in glioma cells transfected with miR-1272 mimic, as assessed by western blotting. (**B**) Correlation analysis between ADAM9 expression and CDCP1 expression in glioma. (**C**) The effect of CDCP1 on the migration ability of SHG44 cells was evaluated by Transwell assay. (**D**) Statistical diagram of the migration results. (**E**) The CCK8 assay was employed to detect the effect of CDCP1 on the proliferation of SHG44 cells. Error bars represent mean ± SEM. ** p < 0.01.

A previous study showed that the ADAM9-CDCP1 signaling pathway plays a role in the progression of lung cancer and ADAM9 enhances CDCP1 expression in lung cancer cell lines [16]. However, the potential roles of CDCP1 and the ADAM9-CDCP1 pathway as a whole in the progression of glioma have not yet been characterized. Here, we detected the expression of ADAM9 and CDCP1 in glioma cells transfected with miR-1272 mimic or ADAM9 overexpression plasmid. In SHG44 cells treated with miR-1272, the expression of CDCP1 was decreased, and ADAM9 rescued the expression of CDCP1 (Figure 6A). This result indicates downregulation of the protein level of CDCP1 in glioma cells transfected with miR-1272 and suggests that it is a downstream target of miR-1272 and ADAM9. Further, correlation analysis revealed a significant association between ADAM9 and CDCP1 in glioma (Figure 6B). Importantly, similar results were not observed in all tumors (Supplementary Figure 3), which highlights the potential importance of ADAM9-CDCP1 signaling in the progression of glioma. Additionally, these results imply that miR-1272 serves as a repressor through the ADAM9-CDCP1 pathway.

In order to examine the potential role of CDCP1 in the tumorigenesis of glioma, CDCP1-overexpressing plasmid and its negative control were used to transfect SHG44 cells (Supplementary Figure 1D). The results of Transwell assay showed that CDCP1 significantly promoted the migration ability of SHG44 cells (Figure 6C, 6D). Moreover, a higher proliferation rate was observed in SHG44 cells transfected with CDCP1 (Figure 6E). Taken together, the above results suggest the possibility that miR-1272 regulates the progression of glioma through the ADAM9-CDCP1 pathway.

### CDCP1 acts as a novel oncogene and is correlated with the prognosis of glioma

**Figure 7:**
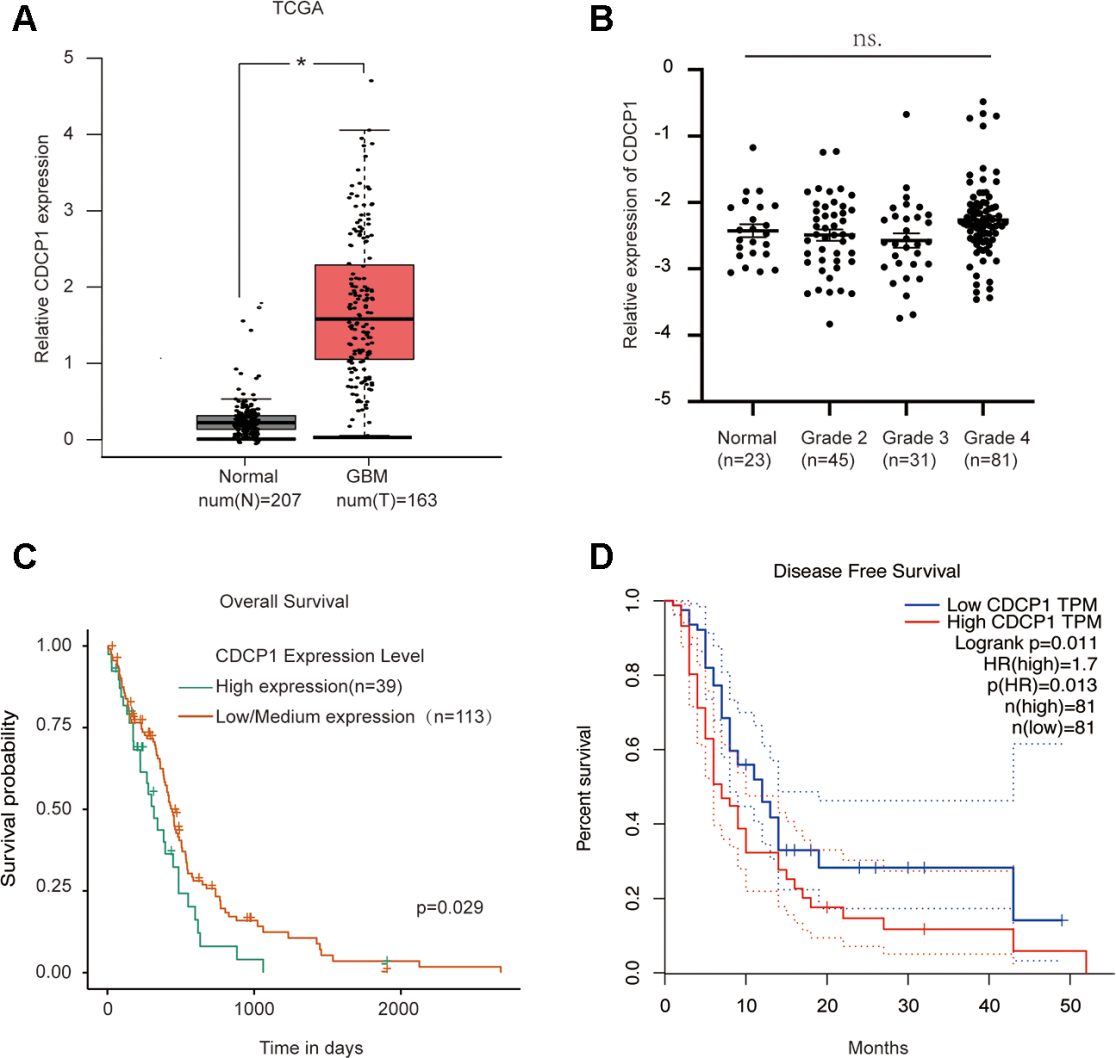
**CDCP1 acts as a novel oncogene and is correlated with glioma prognosis.** (**A**) The expression level of CDCP1 in glioma tissues and normal tissues. (**B**) CDCP1 expression patterns in patients with different glioma grades. (**C** and **D**) The association between CDCP1 expression and glioma prognosis was evaluated by overall survival analysis (**C**) or disease-free survival analysis (**D**). n.s, not significant. Error bars represent mean ± SEM. * p < 0.05.

The above results reveal a novel oncogene, CDCP1, in glioma. To clarify the role of CDCP1 in the progression of glioma, we also analyzed the potential clinical implications of CDCP1 using data from the TCGA database. The results suggested that the expression of CDCP1 was upregulated in glioma tissues (Figure 7A). Correlation analysis revealed no significant association between CDCP1 expression of normal brain tissues and glioma grade among glioma patients (Figure 7B). Further, analysis of overall and disease-free survival among glioma patients revealed that glioma patients with higher expression of CDCP1 had a decreased survival time compared to glioma patients with lower expression of CDCP1 (Figure 7C, 7D). These results suggest that CDCP1 could be regarded as a biomarker for glioma prognosis.

Taken together, these data lead us to believe that reduced miR-1272 expression and increased ADAM9 protein expression are frequent events in human glioma tissues. The negative regulation of ADAM9 by miR-1272 contributes to the functional effects of miR-1272 in the progression of glioma. Then, miR-1272-ADAM9 signaling can regulate the expression of CDCP1, and CDCP1 acts as an oncogene in glioma, thus suggesting that CDCP1 may participate in glioma progression and the miR-1272-ADAM9 signaling pathway.

## DISCUSSION

In recent years, aberrant expression of various miRNAs has been explored in various human tumors, including colorectal cancer, large cell lymphoma, gastric cancer, hepatocellular carcinoma, and lung cancer [17, 18]. Moreover, microRNAs have been found to act as either tumor suppressors [19] or oncogenes [20], owing to microRNA heterogeneity and tumor heterogeneity. In this study, the previously undetermined functional roles of miR-1272 and its target pathway, the ADAM9-CDCP1 pathway, in glioma were investigated.

First, we analyzed the expression of miR-1272 in clinical glioma tissue samples. Our findings suggested that miR-1272 was downregulated in glioma tissues. Subsequent functional experiments showed that overexpression of miR-1272 gave rise to significant decreases in cell proliferation and cell migration, and induced cell cycle arrest. These findings suggest that miR-1272 may function as a tumor suppressor, specifically preventing glioma progression. Further experiments showed that inhibition of miR-1272 had the opposite effects. This further supports the assertion that miR-1272 functions as a tumor suppressor.

In this study, we also demonstrated that ADAM9 is a direct target of miR-1272. We showed this firstly through luciferase reporter assays, which showed that miR-1272 directly recognizes the 3’-UTR of ADAM9 transcripts. Secondly, ADAM9 expression was found to be significantly decreased in glioma cells overexpressing miR-1272. Thirdly, we found that ADAM9 protein and miR-1272 levels exhibited inverse expression patterns in clinical glioma samples. As a direct target of miR-1272, ADAM9 plays an important role in the progression of various glioma cell lines, supporting other studies that have reported that ADAM9 acts as an oncogene and is associated with tumor progression and poorer clinical outcomes.

ADAM9 (A Disintegrin and Metalloproteinase 9) is a cell-surface membrane glycoprotein that is overexpressed in a number of cancers, including gastric cancer [21], lung cancer [16, 22–24], breast cancer [25], prostate cancer [26], and liver cancer [27]. Previous studies have also linked ADAM9 with glioma. In glioma specifically, ADAM9 expression is correlated with poorer clinical outcomes. Similarly, glioblastoma patients have remarkably higher expression of ADAM9 compared to patients with lower-grade gliomas, suggesting that ADAM9 is associated with the glioma severity [28]. Consistent with these studies, our results also revealed increased expression of ADAM9 in glioma samples. Moreover, ADAM9 expression was significantly associated with glioma malignant grade. However, overall survival analysis did not reveal a significant association between the ADAM9 expression and survival, which indicates that high expression of ADAM9 is not significantly associated with overall poorer prognosis in patients diagnosed with glioma.

CDCP1 (CUB-domain-containing protein 1) is a transmembrane protein that serves as a substrate of the Src kinase family. The emerging roles of CDCP1 in various tumors [29–31] led us to evaluate the potential possibility of CDCP1 participating in glioma and miR-1272-induced effects. ADAM9 and CDCP1 were involved in one interaction network, and we noticed that the expression level of full-length CDCP1 was regulated by ADAM9 [16]. The current results indicated that miR-1272 could prohibit the expression of CDCP1 and ADAM9 at the same time. CDCP1 expression was reduced in glioma tissues and was associated with the prognosis of glioma. When considering our results in combination with previous studies [16], we hypothesize that miR-1272 functions as a repressor in glioma through the AMAM9-CDCP1 pathway. However, the functional role of CDCP1 in glioma progression remains elusive. Then, we observed the regulation of glioma cell proliferation and migration by ectopic expression of CDCP1. To the best of our knowledge, this is the first study investigating the functional roles of CDCP1 in glioma. The contribution of the ADAM9-CDCP1-tPA axis and the cleavable form of CDCP1 [16] in glioma requires future exploration.

In summary, we found that a novel tumor-related microRNA, miR-1272, is exceedingly downregulated in glioma tissues, and we identified a link between miR-1272 and ADAM9. Moreover, this study is the first to verify the role of the miR-1272/ADAM9 axis as a regulator of glioma proliferation, migration, and cell cycle progression. We also found that CDCP1 serves as a potential downstream molecule of miR-1272/ADAM9 in glioma. Further study of the miR-1272/ADAM9/CDCP1 axis may provide us with a potential mechanism of glioma progression. Moreover, targeting the miR-1272/ADAM9/CDCP1 pathway may serve as a potential therapeutic strategy for glioma treatment.

## MATERIALS AND METHODS

### Tissue samples and cell lines

Cell lines HA1800, SHG44, U251, T98G, A172, and LN229 purchased from iCell Bioscience Inc. (Shanghai, China) were utilized to perform in vitro experiments. Glioma cells were grown in RMPI 1640 (Hyclone, USA) containing 10% fetal bovine serum (FBS) (Gibco, Invitrogen, Carlsbad, CA, USA), 100 U/ml penicillin, and 100 mg/ml streptomycin. Cells were cultured at 37° C in a humidified atmosphere with 5% CO_2_.

The Department of Neurosurgery at the Affiliated Hospital of Zunyi Medical College (Zunyi, China) provided us with eight human glioma tissues and their adjacent normal tissues. Written informed consent was obtained from patients diagnosed with glioma. This study was approved by the Research Ethics Committee of Zunyi Medical University (Zunyi, Guizhou, China). The TCGA (The Cancer Genome Atlas) database was used for this study (http://cancergenome.nih.gov/). The expression value, sample name, and status (normal or glioblastoma) of each sample were recorded for further analysis of target gene expression.

### Western blotting analysis

Western blot analysis was used to detect the protein expression of the target gene and its downstream molecule. Briefly, transfected cells or tissues were lysed for 30 min at 4° C in RIPA buffer (Beyotime Biotechnology). After centrifuging at 12000 rpm for 15 min, the protein samples were collected and the protein concentration of each group was evaluated using a bicinchoninic acid assay (Thermo Scientific, USA). Protein isolates of glioma cells or tissues were separated by electrophoresis on 10% SDS-polyacrylamide gel, then transferred to polyvinylidene difluoride membranes (Millipore, Beijing, China). Membranes were blocked for 1 h with 5% non-fat dried milk dissolved in 0.05% Tween-20 (TBST) and incubated with primary antibody overnight. An electrochemiluminescence detection system (Thermo Fisher Scientific) was used for signal detection. Immunoblot analysis used the following primary antibodies: anti-ADAM9 (1:1000, A5388, ABclonal), anti-actin antibody (Santa Cruz, Dallas, USA), anti-CDCP1 (1:500, 12754-1-AP, Proteintech, Wuhan, China), and horseradish peroxidase-conjugated goat anti-rabbit IgG H&L secondary antibody (1:10000, ab97080) obtained from Abcam (Cambridge, MA, USA).

### Plasmid construction, oligonucleotides of miR-1272, and transfection

MiR-1272 mimic, negative control miRNA (NC), and miR-1272 inhibitor were chemically designed and synthesized by RiboBio (Guangzhou, Guangdong, China). The ADAM9-overexpression plasmid without 3’UTR was designed and constructed by RiboBio (Guangzhou, Guangdong, China). Cultured glioma cells were transfected with oligonucleotides and plasmids using Lipofectamine 2000 (Invitrogen, Thermo Fisher Scientific, USA), according to the manufacturer’s instructions. Transfection medium without 10% FBS within the glioma cells was replaced by RMPI 1640 medium containing 10% FBS after 6-8 h. Glioma cells transfected with miR-1272 mimic, NC, miR-1272 inhibitor, or ADAM9-overexpression plasmids were harvested 24 or 48 h before further experiments.

### RNA isolation and quantitative real-time PCR (qPCR)

Trizol reagent purchased from Invitrogen was used to isolate the total RNA of cultured glioma cells and tissues. The total RNA concentration was determined by a NanoDrop 1000 spectrophotometer (Thermo Fisher Scientific, Inc., Wilmington, USA). 1 μg RNA of each sample was used for reverse transcription to the cDNAs. The cDNAs were amplified by qRT-PCR using an All-in-OneTM miRNA qRT-PCR Detection Kit (QP015, GeneCopoeia, Rockville, USA). Primers of miR-1272 and U6 were purchased from GeneCopoeia (GeneCopoeia, Rockville, USA). To measure miR-1272 expression, U6 expression was regarded as an endogenous control. The relative expression of miR-1272 was analyzed using the 2^-ΔΔCt^ method.

### Cell Counting Kit-8 assay

Cell proliferation of glioma cells transfected with miR-1272 mimic or inhibitor was assessed using the Cell Counting Kit-8 (CCK-8) assay. Transfected cells were plated in 96-well plates, and then glioma cells were incubated with 10 μL CCK-8 reagent (Dojindo, Tokyo, Japan) and 90 μL RPMI 1640 medium per well. After a 2 h incubation with CCK-8 reagent at 37° C, absorbance (OD) values at 490 nm were assessed by a plate reader and were used to define glioma cell viability (Bio-Tek Instruments, Winooski, VT, USA). The absorbance at each time point (each day over a one-week period) was used to plot the cell viability curve.

### Flow cytometric analysis of the glioma cell cycle

The cultured glioma cells in six-well plates were transfected with ADAM9 plasmids or oligonucleotides and incubated for 48 h. Then, about 1×10^6^ cells derived from each well plate were trypsinized and harvested by centrifugation at 1500 r/minute for 5 min. The harvested cells were washed two times with PBS, collected by centrifugation, fixed with 70% cold ethanol, and stored at 4° C overnight for further analysis. The cells were washed with PBS, exposed to 50 mg/ml of propidium iodide (BD Pharmingen, San Jose, CA, USA), and incubated for 30 min in the dark. The cell cycle property of each group was analyzed by flow cytometry (BD Biosciences, USA).

### Dual luciferase reporter assay

Potential miR-1272-binding sites in the ADAM9 3’-UTR were amplified using PCR from human cDNA, then inserted into the HindIII (aagctt) and Spe I (actagt) sites of a psiCHECK^TM^-2 report plasmid. Glioma cells were cultured in 24-well plates and co-transfected with 3’-UTR luciferase reporters, miR-1272 mimics, or NC. After 48 h of incubation, the luciferase intensity of glioma cells within each group was assessed using a Dual Luciferase Reporter Assay Kit (Promega, Massachusetts, USA), according to the manufacturer’s instructions.

### Cell migration assay

Transwell chambers (BD Bioscience, San Jose, USA) with a diameter of 8 μm were used to evaluate the migration potential of glioma cells. A total volume of 200 μl serum-free RPMI 1640 medium with 1 × 10^4^ transfected glioma cells was added into the upper part of the chamber. As a chemoattractant, 500 μl RMPI 1640 containing 10% FBS was added to the lower part below the chamber. The cells were cultured at 37° C for 12 h or 24 h, and the transfected glioma cells that did not migrate through the membrane of the chamber were wiped off using cotton swabs. The migrated glioma cells were washed twice with PBS, fixed with 500 μL methanol, and stained with 0.1% crystal violet. The number of migrated cells was defined as the average number of cells in five random fields. Stained images of migrated glioma cells were viewed under a microscope.

### Apoptosis assay

An annexin V–fluorescein isothiocyanate/propidium iodide apoptosis detection kit (Beyotime BioTech) was used to measure apoptosis of glioma cells, as described by the manufacturer. The glioma cells were maintained in plates and then transfected with miR-1272 mimics or inhibitors and their negative controls. After incubation for 24 h, the harvested glioma cells were stained with Annexin V-FITC and PI and were then analyzed by flow cytometry using WinMDI 2.9 software (The Scripps Research Institute, La Jolla, CA, USA).

### Data from public online databases

Samples derived from the TCGA database (http://cancergenome.nih.gov/) were used to analyze the differential expression of ADAM9 and CDCP1 between glioma tissues and noncancerous specimens. The clinical information (sample name, expression value, grade stage) was obtained from the Oncomine database [32] (https://www.oncomine.org/resource/login.html). Then, the expression level of ADAM9 and CDCP1 within each grade was identified using this information. The expression value and follow-up time derived from the TCGA database were applied for survival analysis. The expression profiles of ADAM9 and CDCP1 in various tumors were analyzed and acquired from GEPIA [33] (http://gepia.cancer-pku.cn/detail.php?clicktag=matrix).

### Statistical analysis

All experiments were performed in triplicate and all data are presented as mean ± standard error (SE). Differences between the two groups were analyzed by Student’s *t*-test. A one-way ANOVA was used to determine differences among at least three groups. A two-way ANOVA was used to analyze the results of glioma proliferation. Post hoc analyses were performed after one-way or two-way ANOVA. For the cell cycle assay, separate statistical analyses were run for each cell cycle phase (G1, S, G2) using Student’s *t*-test or one-way ANOVA. SPSS 16.0 for Windows was used to perform these analyses (SPSS, IL, USA). Survival analysis was performed according to the Kaplan-Meier method and log-rank test, using GraphPad Prism 5 software. P<0.05 was regarded as a significant difference between groups.

## Supplementary Material

Supplementary Figures
