# Supplementary material for: The emerging role of the MiR-1272-ADAM9-CDCP1 signaling pathway in the progression of glioma
Source: Aging (Albany NY). 2020 Nov 26;13(1):894–909. doi: 10.18632/aging.202196 (PMC7835014; doi:10.18632/aging.202196)
Supplement: Supplementary Figures [file aging-13-202196-s001.pdf]

SUPPLEMENTARY FIGURES

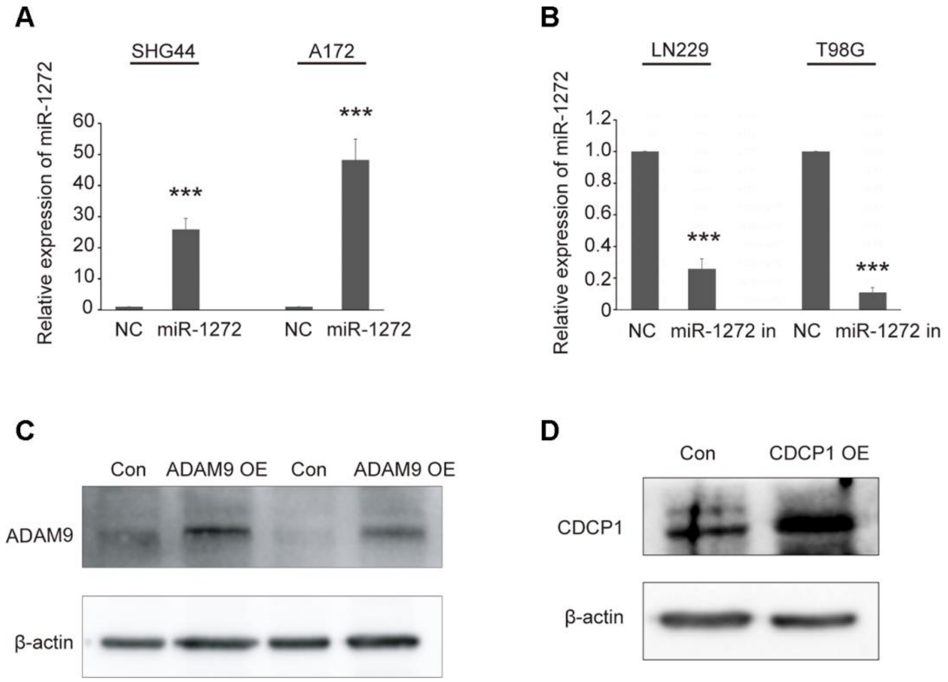

Supplementary Figure 1. Validation of miR-1272, ADAM9, and CDCP1 overexpression or knockdown in glioma cells. (**A**) The ectopic expression of miR-1272 was validated by RT-qPCR. (**B**) MiR-1272 expression was inhibited in LN229 and T98G cells transfected with miR-1272 inhibitor. (**C**) Protein was extracted from A172 cells transfected with ADAM9 overexpressing plasmid and the ADAM9 expression was validated. (**D**) Western blotting was used to detect the overexpression of CDCP1 in SHG44 cells. OE, overexpression. Error bars represent mean ± SEM. *** $p < 0.001$.

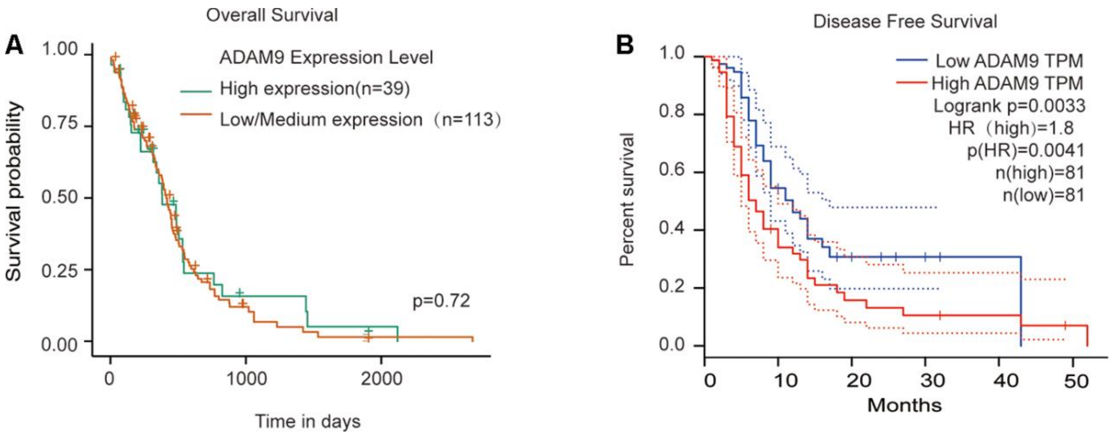

Supplementary Figure 2. Survival analysis of ADAM9 in glioma. The overall survival (**A**) and disease-free survival (**B**) of glioma patients with high ADAM9 expression or low ADAM9 expression using the Kaplan-Meier method.

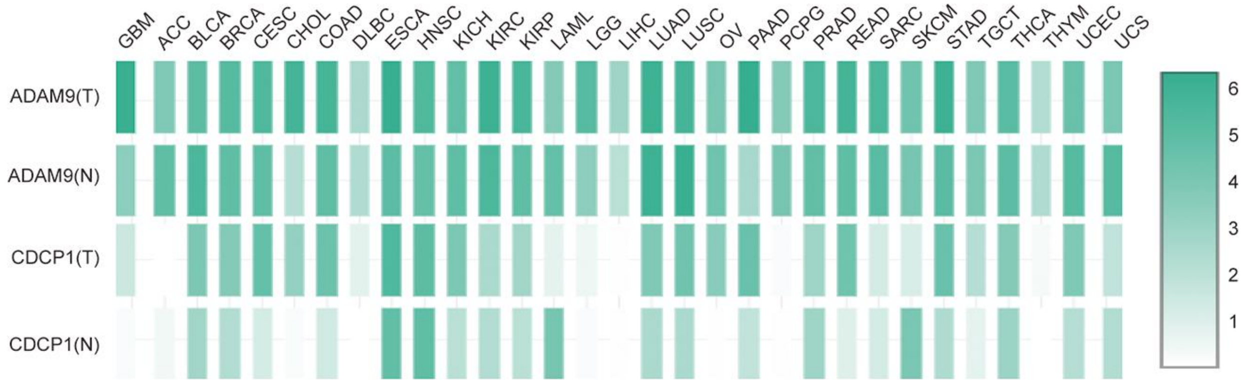

Supplementary Figure 3. Expression patterns of ADAM9 and CDCP1 in various tumors. Relative expression of ADAM9 and CDCP1 in various tumors and normal tissues. Deep color represents high expression of ADAM9 or CDCP1. GBM, Glioblastoma multiforme; ACC, Adrenocortical carcinoma; BLCA, Bladder urothelial carcinoma; BRCA, Breast invasive carcinoma; CESC, Cervical squamous cell carcinoma and endocervical adenocarcinoma; CHOL, Cholangio carcinoma; COAD, Colon adenocarcinoma; DLBC, Lymphoid neoplasm diffuse large B-cell lymphoma; ESCA, Esophageal carcinoma; HNSC, Head and neck squamous cell carcinoma; KICH, Kidney chromophobe; KIRC, Kidney renal clear cell carcinoma; KIRP, Kidney renal papillary cell carcinoma; LAML, Acute myeloid leukemia; LGG, Brain lower grade glioma; LIHC, Liver hepatocellular carcinoma; LUAD, Lung adenocarcinoma; LUSC, Lung squamous cell carcinoma; OV, Ovarian serous cystadenocarcinoma; PAAD, Pancreatic adenocarcinoma; PCPG, Pheochromocytoma and paraganglioma; PRAD, Prostate adenocarcinoma; READ, Rectum adenocarcinoma; SARC, Sarcoma; SKCM, Skin cutaneous melanoma; STAD, Stomach adenocarcinoma; TGCT, Testicular germ cell tumors; THCA, Thyroid carcinoma; THYM, Thymoma; UCEC, Uterine corpus endometrial carcinoma; UCS, Uterine carcinosarcoma.
